# 10-(Prop-2-yn­yl)-10*H*-phenothia­zine

**DOI:** 10.1107/S1600536810040638

**Published:** 2010-10-20

**Authors:** Younas Aouine, Anouar Alami, Abdelilah El Hallaoui, Abdelrhani Elachqar, Hafid Zouihri

**Affiliations:** a Laboratoire de Chimie Organique, Faculté des Sciences, Dhar el Mahraz, Université Sidi Mohammed Ben Abdellah, Fès, Morocco; b Laboratoires de Diffraction des Rayons X, Centre National pour la Recherche Scientifique et Technique, Angle Allal, AlFassi et Avenue des FAR, Hay Ryad, BP 8027, Rabat, Morocco

## Abstract

In the mol­ecule of the title compound, C_15_H_11_NS, the butterfly angle between the two planes defined by the two wings of the phenothia­zine unit is 33.5 (8)°. The dihedral angles between the two benzene rings and the propynyl group are 85 (4) and 63 (4)°.

## Related literature

For the 1,3-dipolar addition reaction in chemical synthesis, see: Kumar *et al.* (2006 ▶); Kalita *et al.* (2006 ▶); Sibi *et al.* (2006 ▶); Choi *et al.* (2006 ▶); Ji-Cai *et al.* (2007 ▶); Aouine *et al.* (2008 ▶).
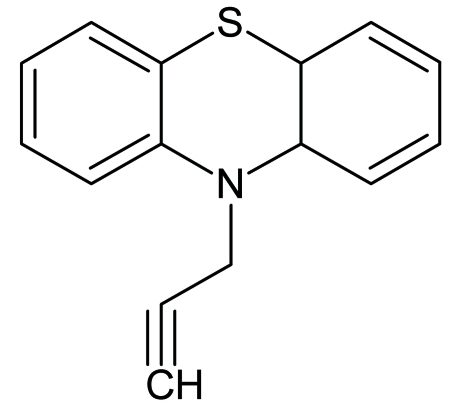


## Experimental

### 

#### Crystal data

C_15_H_11_NS
*M* *_r_* = 237.31
Monoclinic, 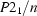

*a* = 10.5306 (10) Å
*b* = 7.2981 (6) Å
*c* = 15.6782 (14) Å
β = 96.023 (3)°
*V* = 1198.27 (18) Å^3^
*Z* = 4
Mo *K*α radiation
μ = 0.24 mm^−1^
*T* = 296 K
0.42 × 0.38 × 0.17 mm

#### Data collection

Bruker APEXII CCD detector diffractometer
16905 measured reflections
3688 independent reflections
2683 reflections with *I* > 2σ(*I*)
*R*_int_ = 0.034

#### Refinement

*R*[*F* ^2^ > 2σ(*F* ^2^)] = 0.046
*wR*(*F* ^2^) = 0.151
*S* = 1.08
3688 reflections
155 parameters
H-atom parameters constrained
Δρ_max_ = 0.35 e Å^−3^
Δρ_min_ = −0.24 e Å^−3^

### 

Data collection: *APEX2* (Bruker, 2005 ▶); cell refinement: *SAINT* (Bruker, 2005 ▶); data reduction: *SAINT*; program(s) used to solve structure: *SHELXS97* (Sheldrick, 2008 ▶); program(s) used to refine structure: *SHELXL97* (Sheldrick, 2008 ▶); molecular graphics: *PLATON* (Spek, 2009 ▶); software used to prepare material for publication: *publCIF* (Westrip, 2010 ▶).

## Supplementary Material

Crystal structure: contains datablocks I, global. DOI: 10.1107/S1600536810040638/ds2063sup1.cif

Structure factors: contains datablocks I. DOI: 10.1107/S1600536810040638/ds2063Isup2.hkl

Additional supplementary materials:  crystallographic information; 3D view; checkCIF report

## Figures and Tables

**Fig. 1.**
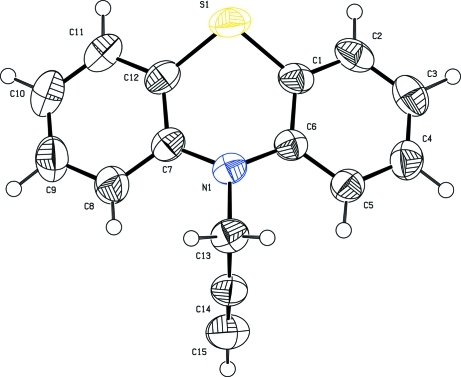
ORTEP of the molecule of the title compound showing the atom-labelling scheme and 50% probability displacement ellipsoids.

**Fig. 2.**
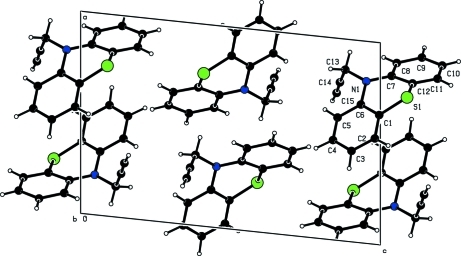
Partial packing view.

## supplementary crystallographic information

### Comment

The 1,3-dipolar addition reaction as a versatile method for preparing five-membered heterocyclic compounds is a classical reaction in organic chemistry and has been studied extensively. These cycloadditions have been utilized for the preparation of compounds that are fundamental importance in diverse fields of chemistry, see: Kumar *et al.* (2006); Kalita *et al.* (2006); Sibi *et al.* (2006); Choi *et al.* (2006); Ji-Cai *et al.* (2007). This approach consists of preparing firstly, heterocyclic dipolarophiles by nucleophilic substitution of propargyl bromide with heterocyclic compounds, see: Y. Aouine *et al.* (2008). The dipolarophile 10-(prop-2-ynyl)-10*H*-phenothiazine was obtained with good yield.

In the molecule of the title compound, C_15_H_11_NS, the butterfly angle between the two planes defined by the two wings of the phenothiazine unit is 33.5 (8)°. The dihedral angles between the two phenyls and the propynyl are: 85 (4)° and 63 (4)°, respictively.

### Experimental

To a stirred solution (10 mmoles) of phenothiazine, potassium carbonate (15 mmoles) and a catalytic amount of tetrabutylammonium bromide in 10 ml of dry acetone, 10 mmoles of propargyl bromide was added. The mixture was stirred at room temperature for 6 h. The solvent was evaporated under vacuum and the residue was extracted with ether. The organic layer was washed with water, dried with sodium sulfate (Na2SO4), and the solvent was removed. The product was purified by column chromatography on silica gel using ether/hexane as eluant to afford pure alkyne. The purity of the compound was checked by determining its melting point (82–84°C). Suitable single-crystal of the title compound was obtained by recrystallization from ethanol. The structure of the product was established on the basis of NMR spectroscopy (1*H*, 13 C) and MS data.

### Refinement

All H atoms were fixed geometrically and treated as riding with C—H = 0.97 Å (methyne) and 0.93Å (aromatic) with *U*_iso_(H) = 1.2Ueq(C).

### Crystal data

**Table:**

C_15_H_11_NS	*F*(000) = 496
*M**_r_* = 237.31	*D*_x_ = 1.315 Mg m^−^^3^
Monoclinic, *P*2_1_/*n*	Melting point: 355 K
Hall symbol: -P 2yn	Mo *K*α radiation, λ = 0.71073 Å
*a* = 10.5306 (10) Å	Cell parameters from 2646 reflections
*b* = 7.2981 (6) Å	θ = 1.7–26.2°
*c* = 15.6782 (14) Å	µ = 0.24 mm^−^^1^
β = 96.023 (3)°	*T* = 296 K
*V* = 1198.27 (18) Å^3^	Block, colourless
*Z* = 4	0.42 × 0.38 × 0.17 mm

### Data collection

**Table:**

Bruker APEXII CCD detector diffractometer	2683 reflections with *I* > 2σ(*I*)
Radiation source: fine-focus sealed tube	*R*_int_ = 0.034
graphite	θ_max_ = 30.6°, θ_min_ = 2.2°
ω and φ scans	*h* = −15→15
16905 measured reflections	*k* = −10→10
3688 independent reflections	*l* = −21→22

### Refinement

**Table:**

Refinement on *F*^2^	Secondary atom site location: difference Fourier map
Least-squares matrix: full	Hydrogen site location: inferred from neighbouring sites
*R*[*F*^2^ > 2σ(*F*^2^)] = 0.046	H-atom parameters constrained
*wR*(*F*^2^) = 0.151	*w* = 1/[σ^2^(*F*_o_^2^) + (0.0829*P*)^2^ + 0.0989*P*] where *P* = (*F*_o_^2^ + 2*F*_c_^2^)/3
*S* = 1.08	(Δ/σ)_max_ = 0.002
3688 reflections	Δρ_max_ = 0.35 e Å^−^^3^
155 parameters	Δρ_min_ = −0.24 e Å^−^^3^
0 restraints	Extinction correction: *SHELXL97* (Sheldrick, 2008), Fc^*^=kFc[1+0.001xFc^2^λ^3^/sin(2θ)]^-1/4^
Primary atom site location: structure-invariant direct methods	Extinction coefficient: 0.015 (3)

### Special details

**Table:**

Geometry. All e.s.d.'s (except the e.s.d. in the dihedral angle between two l.s. planes) are estimated using the full covariance matrix. The cell e.s.d.'s are taken into account individually in the estimation of e.s.d.'s in distances, angles and torsion angles; correlations between e.s.d.'s in cell parameters are only used when they are defined by crystal symmetry. An approximate (isotropic) treatment of cell e.s.d.'s is used for estimating e.s.d.'s involving l.s. planes.
Refinement. Refinement of *F*^2^ against ALL reflections. The weighted *R*-factor *wR* and goodness of fit *S* are based on *F*^2^, conventional *R*-factors *R* are based on *F*, with *F* set to zero for negative *F*^2^. The threshold expression of *F*^2^ > σ(*F*^2^) is used only for calculating *R*-factors(gt) *etc*. and is not relevant to the choice of reflections for refinement. *R*-factors based on *F*^2^ are statistically about twice as large as those based on *F*, and *R*- factors based on ALL data will be even larger.

### Fractional atomic coordinates and isotropic or equivalent isotropic displacement parameters (Å^2^)

**Table:**

	*x*	*y*	*z*	*U*_iso_*/*U*_eq_	
S1	0.74384 (5)	1.23619 (6)	1.08918 (3)	0.06194 (18)	
C1	0.64205 (14)	1.18256 (18)	0.99624 (9)	0.0453 (3)	
C12	0.81567 (13)	1.0206 (2)	1.10752 (9)	0.0476 (3)	
C6	0.68419 (12)	1.06413 (17)	0.93508 (8)	0.0409 (3)	
C7	0.84191 (12)	0.9120 (2)	1.03762 (8)	0.0444 (3)	
C5	0.60793 (14)	1.0439 (2)	0.85724 (9)	0.0482 (3)	
H5	0.6338	0.9661	0.8154	0.058*	
C4	0.49414 (14)	1.1390 (2)	0.84205 (11)	0.0572 (4)	
H4	0.4464	1.1286	0.7889	0.069*	
C3	0.45030 (16)	1.2484 (2)	0.90383 (12)	0.0594 (4)	
H3	0.3724	1.3087	0.8935	0.071*	
C14	0.78307 (14)	0.6913 (2)	0.86512 (9)	0.0512 (3)	
C2	0.52371 (17)	1.26762 (19)	0.98158 (11)	0.0545 (4)	
H2	0.4937	1.3382	1.0246	0.065*	
C13	0.84863 (13)	0.8676 (2)	0.88310 (9)	0.0504 (3)	
H13A	0.9391	0.8445	0.8970	0.060*	
H13B	0.8386	0.9419	0.8315	0.060*	
C8	0.90857 (15)	0.7493 (2)	1.05489 (11)	0.0582 (4)	
H8	0.9266	0.6740	1.0099	0.070*	
C10	0.91968 (17)	0.8038 (3)	1.20672 (11)	0.0690 (5)	
H10	0.9457	0.7676	1.2627	0.083*	
C11	0.85181 (15)	0.9640 (3)	1.19087 (10)	0.0585 (4)	
H11	0.8301	1.0346	1.2365	0.070*	
C15	0.72916 (18)	0.5503 (3)	0.85403 (11)	0.0663 (4)	
H15	0.6866	0.4391	0.8453	0.080*	
C9	0.94833 (17)	0.6984 (3)	1.13868 (12)	0.0707 (5)	
H9	0.9952	0.5912	1.1490	0.085*	
N1	0.80200 (10)	0.97117 (16)	0.95310 (7)	0.0436 (3)	

### Atomic displacement parameters (Å^2^)

**Table:**

	*U*^11^	*U*^22^	*U*^33^	*U*^12^	*U*^13^	*U*^23^
S1	0.0868 (3)	0.0449 (2)	0.0533 (3)	−0.00690 (19)	0.0031 (2)	−0.01417 (16)
C1	0.0585 (8)	0.0314 (6)	0.0470 (7)	−0.0052 (5)	0.0096 (6)	0.0007 (5)
C12	0.0450 (6)	0.0521 (8)	0.0449 (7)	−0.0122 (6)	0.0006 (5)	−0.0051 (6)
C6	0.0473 (6)	0.0334 (6)	0.0426 (6)	−0.0073 (5)	0.0075 (5)	0.0020 (5)
C7	0.0374 (6)	0.0507 (8)	0.0447 (7)	−0.0080 (5)	0.0033 (5)	−0.0021 (6)
C5	0.0555 (8)	0.0423 (7)	0.0463 (7)	−0.0043 (6)	0.0027 (6)	0.0008 (6)
C4	0.0575 (8)	0.0522 (9)	0.0595 (9)	−0.0032 (7)	−0.0053 (7)	0.0091 (7)
C3	0.0560 (8)	0.0437 (8)	0.0788 (11)	0.0036 (6)	0.0088 (8)	0.0134 (7)
C14	0.0540 (8)	0.0558 (9)	0.0452 (7)	0.0055 (7)	0.0118 (6)	−0.0064 (6)
C2	0.0685 (9)	0.0341 (7)	0.0638 (9)	0.0034 (6)	0.0202 (8)	0.0038 (6)
C13	0.0456 (7)	0.0608 (9)	0.0469 (7)	−0.0033 (6)	0.0150 (6)	−0.0047 (6)
C8	0.0486 (7)	0.0684 (11)	0.0566 (9)	0.0095 (7)	0.0013 (6)	−0.0029 (7)
C10	0.0587 (9)	0.0943 (14)	0.0512 (9)	0.0004 (9)	−0.0072 (7)	0.0113 (9)
C11	0.0553 (8)	0.0759 (11)	0.0434 (7)	−0.0118 (8)	0.0008 (6)	−0.0050 (7)
C15	0.0830 (12)	0.0568 (10)	0.0602 (10)	−0.0058 (9)	0.0134 (8)	−0.0081 (8)
C9	0.0587 (9)	0.0832 (12)	0.0681 (11)	0.0157 (9)	−0.0038 (8)	0.0121 (10)
N1	0.0435 (6)	0.0475 (6)	0.0402 (6)	−0.0038 (5)	0.0066 (4)	−0.0041 (5)

### Geometric parameters (Å, °)

**Table:**

S1—C12	1.7563 (17)	C3—H3	0.9300
S1—C1	1.7599 (15)	C14—C15	1.179 (2)
C1—C2	1.390 (2)	C14—C13	1.473 (2)
C1—C6	1.3974 (19)	C2—H2	0.9300
C12—C11	1.385 (2)	C13—N1	1.4594 (16)
C12—C7	1.4034 (19)	C13—H13A	0.9700
C6—C5	1.3965 (19)	C13—H13B	0.9700
C6—N1	1.4162 (17)	C8—C9	1.387 (2)
C7—C8	1.391 (2)	C8—H8	0.9300
C7—N1	1.4151 (17)	C10—C9	1.374 (3)
C5—C4	1.384 (2)	C10—C11	1.379 (3)
C5—H5	0.9300	C10—H10	0.9300
C4—C3	1.372 (2)	C11—H11	0.9300
C4—H4	0.9300	C15—H15	0.9300
C3—C2	1.380 (3)	C9—H9	0.9300
			
C12—S1—C1	98.67 (7)	C3—C2—H2	119.7
C2—C1—C6	120.44 (14)	C1—C2—H2	119.7
C2—C1—S1	119.69 (12)	N1—C13—C14	114.10 (11)
C6—C1—S1	119.73 (11)	N1—C13—H13A	108.7
C11—C12—C7	120.73 (15)	C14—C13—H13A	108.7
C11—C12—S1	119.52 (12)	N1—C13—H13B	108.7
C7—C12—S1	119.66 (11)	C14—C13—H13B	108.7
C5—C6—C1	118.04 (13)	H13A—C13—H13B	107.6
C5—C6—N1	122.35 (12)	C9—C8—C7	120.59 (16)
C1—C6—N1	119.61 (12)	C9—C8—H8	119.7
C8—C7—C12	117.82 (14)	C7—C8—H8	119.7
C8—C7—N1	122.48 (13)	C9—C10—C11	119.11 (16)
C12—C7—N1	119.70 (13)	C9—C10—H10	120.4
C4—C5—C6	120.38 (14)	C11—C10—H10	120.4
C4—C5—H5	119.8	C10—C11—C12	120.58 (16)
C6—C5—H5	119.8	C10—C11—H11	119.7
C3—C4—C5	121.32 (15)	C12—C11—H11	119.7
C3—C4—H4	119.3	C14—C15—H15	180.0
C5—C4—H4	119.3	C10—C9—C8	121.09 (18)
C4—C3—C2	118.93 (15)	C10—C9—H9	119.5
C4—C3—H3	120.5	C8—C9—H9	119.5
C2—C3—H3	120.5	C7—N1—C6	120.06 (10)
C15—C14—C13	177.31 (16)	C7—N1—C13	117.11 (12)
C3—C2—C1	120.68 (15)	C6—N1—C13	117.06 (11)
